# Application of the Artificial Intelligence Algorithm Model for Screening of Inborn Errors of Metabolism

**DOI:** 10.3389/fped.2022.855943

**Published:** 2022-05-19

**Authors:** Muping Zhou, Liyuan Deng, Yan Huang, Ying Xiao, Jun Wen, Na Liu, Yingchao Zeng, Hua Zhang

**Affiliations:** Neonatal Disease Screening Center, The Maternal and Child Health Hospital of Shaoyang City, Shaoyang, China

**Keywords:** artificial intelligence, inborn errors of metabolism, newborn screening, incidence rate, tandem mass spectrometry

## Abstract

Inborn errors of metabolism (IEMs) are strongly related to abnormal growth and development in newborns and can even result in death. In total, 94,648 newborns were enrolled for expanded newborn screening using tandem mass spectrometry (MS/MS) from 2016 to 2020 at the Neonatal Disease Screening Center of the Maternal and Child Health Hospital in Shaoyang City, China. A total of 23 confirmed cases were detected in our study with an incidence rate of 1:4,115. A total of 10 types of IEM were identified, and the most common IEMs were phenylalanine hydroxylase deficiency (PAHD; 1:15,775) and primary carnitine deficiency (PCD; 1:18,930). Mutations in phenylalanine hydroxylase (PAH) and SLC22A5 were the leading causes of IEMs. To evaluate the application effect of artificial intelligence (AI) in newborn screening, we used AI to retrospectively analyze the screening results and found that the false-positive rate could be decreased by more than 24.9% after using AI. Meanwhile, a missed case with neonatal intrahepatic cholestasis citrin deficiency (NICCD) was found, the infant had a normal citrulline level (31 μmol/L; cutoff value of 6–32 μmol/L), indicating that citrulline may not be the best biomarker of intrahepatic cholestasis citrin deficiency. Our results indicated that the use of AI in newborn screening could improve efficiency significantly. Hence, we propose a novel strategy that combines expanded neonatal IEM screening with AI to reduce the occurrence of false positives and false negatives.

## Introduction

Inborn errors of metabolism (IEMs) refer to a class of diseases in which genetic mutations cause enzyme defects, membrane dysfunction, or receptor defects, leading to a series of clinical symptoms caused by the accumulation of intermediate, bypass metabolites, or terminal metabolite deficiency (1–4). To date, more than 1,400 IEMs have been described (5). The prevalence of individual IEM is low, but the overall incidence rate is high. IEMs pose a great threat to the development of individuals, families, and society (6). Therefore, early presymptomatic screening, diagnosis, and treatment are important to reduce the incidence of IEMs.

The application of tandem mass spectrometry (MS/MS) in newborn screening enabled a robust increase in the screening and detection of IEMs and made it a highly efficient and economical program. Nowadays, newborn screening for IEMs by MS/MS is being widely performed worldwide; however, many problems remain. First, the level of metabolites in neonatal blood is affected by many factors, such as the neonatal physiological state, maternal factors, the time of day when the blood is collected, and sample quality. Moreover, neonatal screening is complicated due to the complex disease-specific indicators and different criteria used by doctors. All these factors increase the risk of false positives and false negatives in newborn screening (7–11). Screening results for primary carnitine deficiency (PCD; OMIM# 212,140) and methylmalonic acidemia (MMA; OMIM #251,000, 277,400, 277,410, 251,100, 251,110, 277,380, 309,541, 613,646, 614,265, and 614,857) have a high false-positive rate that necessitate recall and follow-up with suspected positives, which can be difficult (12, 13). Some screening indicators of IEMs such as citrulline, an indicator of neonatal intrahepatic cholestasis citrin deficiency (NICCD; OMIM #605,814), have a lower specificity and higher false-positive rates (14). Thus, there is an urgent clinical need to improve the neonatal screening efficiency of IEMs, researchers are making efforts to this end, and some gratifying research results have been achieved. Yosuke developed a second-tier liquid chromatography- (LC-) MS/MS analysis method to minimize false-positive cases in newborn screening by MS/MS (15). Their works could effectively decrease the false-positive rate of glutaric acidemia type I (GA-I), 3-methylcrotonyl-CoA carboxylase deficiency (3-MCCD), and were useful for differential diagnosis in cases positive for C5-OHacylcarnitine or C5-acylcarnitine. In another research reported by Lin et al. (16), they designed a high-throughput iPLEX genotyping assay to detect NICCD in the Chinese population. Newborns with citrulline levels between 1/2 cutoff and cutoff values of the upper limit were recruited for this assay (29,364 out of 237,630), and five missed cases were finally found. Combining newborn metabolic screening with genetic screening could greatly improve the performance of the current newborn screening program. Although a number of researchers have proposed effective protocols to improve screening efficiency, the protocols need an additional test, which would increase reporting time and the cost for parents. We applied an artificial intelligence (AI) disease risk assessment model to neonatal IEMs to explore a new strategy to reduce the occurrence of false positives and false negatives in newborn screening.

## Materials and Methods

### Clinical Data

A retrospective study was conducted on 94,648 neonates and 23 confirmed cases from 2016 to 2020 at the Maternal and Child Health Hospital of Shaoyang City, China.

### Blood Collection

Blood samples were collected *via* heel prick from breastfed newborns 72 h after birth, blotted on specialized filter papers, dried at low temperatures, and delivered to the Shaoyang Neonatal Screening Center.

### Newborn Screening by Tandem Mass Spectrometry

Dried blood spots were pretreated according to the NeoBase™ non-derivatized MS/MS kit (PerkinElmer, Turku, Finland) with an organic solvent containing the internal amino acid and carnitine standards. All samples were analyzed using a Waters ACQUITY TQD MS/MS screening system. After passing internal quality control, data analysis, and report distribution were performed.

### Genetic Testing

Genomic DNA was extracted from peripheral blood (or dried blood spots) of presumptive patients using the Qiagen Mini Blood DNA kit (Hilden, Germany). The capture probes of genes related to IEMs were customized by Agilent (Palo Alto, CA, United States), and targeted genomic DNA region sequences were enriched by multiple probe hybridization. After extraction and library preparation of the targeted region sequences, DNA samples of probands were sequenced on the HiSeq2500 (Illumina, San Diego, CA, United States) platform. All identified variants were validated using Sanger sequencing on an ABI 3500XL (SCIEX, Boston, MA, United States).

### Data Collection and Standardization

The following data were collected:

(1) Laboratory background information included instrument model, reagent type, quality control type, laboratory quality control rules, punched spot size, filter paper type, cold chain transportation, blood collection needle type, cutoff value range, and positive rules.
(2) Quality control data included quality control number, quality control type, batch number, amino acid internal standard number, acylcarnitine internal standard batch number, delivery time, test time, and test value of the quality control analyte.
(3) Screening test data included screening number, the age of mother, gestational week, gestational number, fetal age, hyperthyroidism, consuming antithyroid medication or not, lactation mode, residence of mother, breast-feed times, sex, birth date, birth weight, initial screening result, reexamined result, sample number, screening times, blood collection time, delivery date, experiment date, experimental method, quality control number, and the concentration of analytes.
(4) Confirmed cases included the screening number, confirmed disorder, urine organic acid test result, blood ammonia test result, blood gas analysis result, blood routine test result, liver function test result, vitamin B12 detection result, imaging, gene detection result, and other test results.
(5) Standardized median multiple (multiple of the medium, MoM) methods were applied in which the median of the original concentration divided by the biochemical indicators were applied to the detection indicators to eliminate the influence of regional and laboratory differences. We then trained the disease model by combining the MoM, gestational week, neonatal blood collection interval, neonatal weight, and corresponding IEMs.

### The Artificial Intelligence Disease Risk Assessment Model of Inborn Errors of Metabolism

The AI disease risk assessment model for IEMs was developed by Zhejiang Biosan Biochemical Technologies Co., Ltd. (17, 18), and the construction process of AI model was as follows:

(1) *Construction and selection of model indicators*: core indicators were selected by information gain and correlation coefficient, and combined feature construction is performed.
(2) *Model selection and training*: the model training phase divides the screening data into the training set and test set in the ratio of 8:2, and the best performing model is selected by integrating learning models such as random forest, gradient boosting tree algorithm, and artificial neural network algorithm for training.
(3) *Model evaluation*: the model firstly satisfied the identification rate of 100% for positive cases, and then the false-positive rate was compared to select the optimal training model. In addition, in the risk judgment process, the machine learning model performed risk prediction for the test samples, predicted the risk of samples suffering from different inherited metabolic diseases, converted them into scores from 0 to 100 by the risk value mapping algorithm, and set different risk judgment cutoff values according to the risk value scores and the prevalence of the disease at each location.

After standardization and selection, approximately 3.67 million screening samples and more than 3,000 confirmed cases were used to train the model. The random forest algorithm performed the best in the evaluation, and the AI model was constructed by the random forest algorithm finally. The AI disease panel is presented in Table 1.

**TABLE 1:** Panel of the artificial intelligence (AI) disease risk assessment model.

IEMs (OMIM code)	Abbreviation	IEMs (OMIM code)	Abbreviation
Argininosuccinic aciduria (#207,900)	ASA	Methylmalonic acidemia (#251,000, #251,100, #251,110, #613, 646, and #614, 265)	MMA
Beta-ketothiolase deficiency (#203,750)	BKD	Methylmalonic aciduria combined with homocystinuria (#277,400, #277,410, #277,380, #309,541 and #614, 857)	MMA-HCY
Carnitine palmitoyltransferase I deficiency (#255,120)	CPT-ID	Neonatal intrahepatic cholestasis citrin deficiency (#605,814)	NICCD
Citrullinemia type I (#215,700)	CIT-I	Ornithine transcarbamylase deficiency (#311,250)	OTCD
Glutaric acidemia I (#231,670)	GA-I	Phenylalanine hydroxylase deficiency (#261,600)	PAHD
Multiple acyl-CoA dehydrogenase deficiency (#231,680)	MADD	Primary carnitine deficiency (#212,140)	PCD
Holocarboxylase synthetase deficiency (#253, 270)	HCSD	Propionic acidemia (#606,054)	PA
Homocystinuria (#236,200)	HCY	Short-chain acyl-CoA dehydrogenase deficiency (#201,470)	SCADD
Hypermethioninemia (#250,850)	H-MET	Tetrahydrobiopterin deficiency (#233,910, #261,640, #612,716, #264,070, and #261,630)	BH4D
Hyperprolinuria (#239,500)	H-PRO	Very-long-chain acyl-CoA dehydrogenase deficiency (#201,475)	VLCADD
Isobutyryl-CoA dehydrogenase deficiency (#611,283)	IBDD	3-hydroxy-3-methylglutaryl-CoA lyase deficiency (#246,450)	3-HMGD
Isovaleric acidemia (#243,500)	IVA	2-methylbutyryl-CoA dehydrogenase deficiency (#610,006)	2-MBDD
Maple syrup urine disease (#248,600)	MSUD	3-methylcrotonyl-CoA carboxylase deficiency (#210,200 and #210,210)	3-MCCD
Medium-chain acyl-CoA dehydrogenase deficiency (#201,450)	MCADD		

### Risk Assessment and Artificial Intelligence Disease Model Verification

**FIGURE 1:**
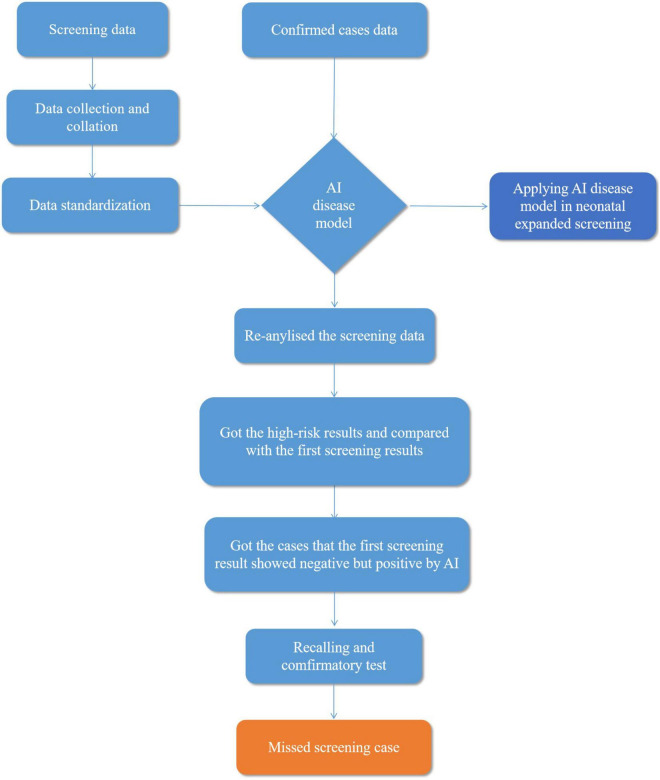
Flow diagram explaining artificial intelligence (AI) disease model applied in neonatal screening.

Preliminary screening data were imported into the system as training data for model learning, according to the standard screening data, outputted to the high- and low-risk sample lists, and then compared with the initial screening results judged by the clinician. The performance of the AI disease model was evaluated through the comparison results between AI and physicians. The flow diagram is shown in Figure 1.

During the risk determination process, the machine learning model performed risk prediction on the test samples to develop different IEMs, converted them into scores from 0 to 100 using the risk value mapping algorithm, and set different risk determination cutoff values according to the risk value scores and the incidence of IEMs. In addition, the sample input machine learning model directly output the probability value of IEM through the probability value of the analogy model and the actual population risk value to guarantee the rationality of the risk cutoff value. The probability value of the sample was mapped to the risk value of the model population in the sample risk prediction, and the quantile of the sample probability value in the population was calculated. A higher quantile indicates a higher risk of the disorder, so the IEM AI diagnosis platform was judged by the risk of different IEMs.

### Ethics Statements

The study was reviewed and approved by the Ethics Committee of the Maternal and Child Health Hospital in Shaoyang City. Written informed consent to participate in this study was provided by the participant’s legal guardian/next of kin.

## Results

### Newborn Screening

**FIGURE 2:**
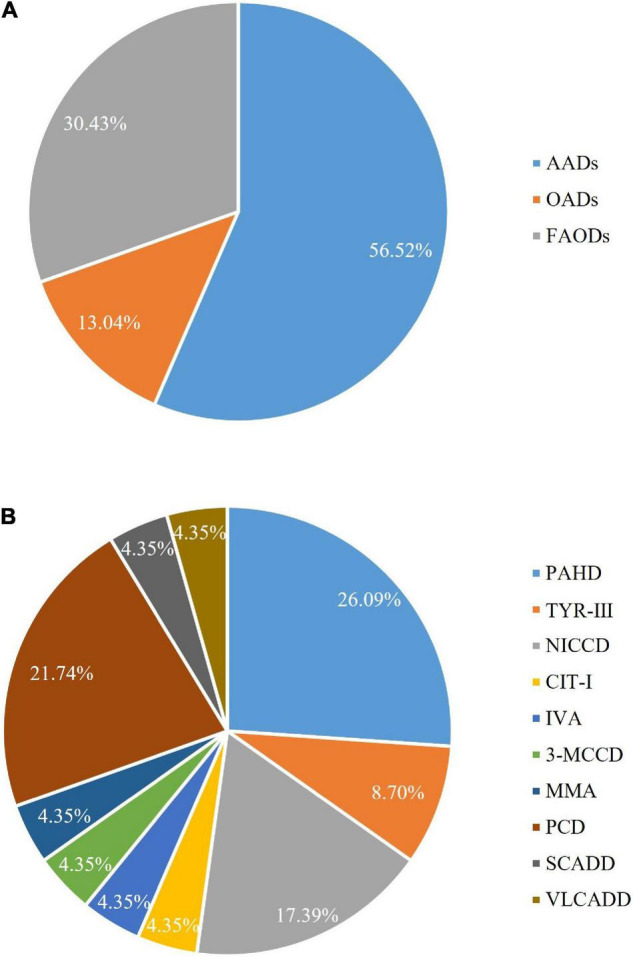
Proportion of inborn errors of metabolism (IEMs) in Shaoyang area from 2016 to 2020.

A total of 94,648 newborns were screened in the Shaoyang region from 2016 to 2020, of which 1,988 were initially screened positive with a recall rate of 2.1%, and 23 were confirmed positive (10 men and 13 women; Table 2 and Figure 2). Out of the 23 confirmed cases, one patient was found to have both NICCD and citrullinemia type I (CIT-I). The overall incidence rate was 1:4,115 (Table 2).

**TABLE 2:** Newborn screening of the Shaoyang area from 2016 to 2020.

Year	Number of screenings	Suspected positive cases	Confirmed cases	Frequency
2016	5,016	90	3	1:1,672
2017	10,717	201	4	1:2,679
2018	18,743	378	4	1:4,686
2019	22,447	522	5	1:4,489
2020	37,725	797	7	1:5,389
Total	94,648	1,988	23	1:4,115

### Laboratory Test and Gene Analysis of Children With Positive Diagnosis

There were six cases of phenylalanine hydroxylase deficiency (PAHD, OMIM# 261,600), five cases of PCD, four cases of NICCD (one case was found by the AI disease model, which was missed by the initial screen), two cases of tyrosinemia type III (TYR-III; OMIM #276,710), one case each of CIT-I (OMIM #215,700), methylmalonic aciduria combined with homocystinuria (MMA-HCY; OMIM#277,400), short-chain acyl-CoA dehydrogenase deficiency (SCAD; OMIM# 201,470), isovaleric acidemia (IVA; OMIM #243,500), 3-MCCD (OMIM #243,500), and very-long-chain acyl-CoA dehydrogenase deficiency (VLCADD; OMIM #201,475; Table 3).

**TABLE 3:** Incidence rate of inborn errors of metabolism (IEMs).

Disorders (OMIM code)	Confirmed cases	Frequency
**Amino acid disorders**	**13**	1:7,281
Phenylalanine hydroxylase deficiency (#261,600)	6	1:15,775
Citrullinemia type I (#215,700)	1	1:94,648
Neonatal intrahepatic cholestasis citrin deficiency (#605,814)	4	1:23,662
Tyrosinemia type III (#276,710)	2	1:47,324
**Organic acid disorders**	**3**	**1:31,549**
Isovaleric acidemia (#243,500)	1	1:94,648
Methylmalonic aciduria combined with homocystinuria (#277,400)	1	1:94,648
3-methylcrotonyl-CoA carboxylase deficiency (#210,200)	1	1:94,648
**Fatty acid oxidation disorders**	**7**	**1:13,521**
Primary carnitine deficiency (#212,140)	5	1:18,930
Short-chain acyl-CoA dehydrogenase deficiency (#201,470)	1	1:94,648
Very-long-chain acyl-CoA dehydrogenase deficiency (#201,475)	1	1:94,648
Total	23	1:4,115

*The bold values mean all the IEMs are classified as amino acid disorders, organic acid disorders, and fatty acid oxidation disorders.*

Of the 94,648 newborn screening samples, 10 types of IEM were identified in 23 cases, including four types (13 cases) of amino acid disorder (AAD), which accounted for 56.52%, with an incidence rate of 1:7,281; three types (three cases) of organic acid disorder (OAD), which accounted for 13.04%, with an incidence rate of 1:31,549; and three types (seven cases) of fatty acid oxidation disorder (FAOD), which accounted for 30.43%, with an incidence rate of 1:13,521 (Figure 2 and Table 3). The highest incidence rates of IEMs were PAHD (1:15,775), PCD (1:18,930), and NICCD (1:23,662). The incidence of each disorder is shown in Table 3. The genetic diagnosis results of 23 confirmed cases are shown in Table 4, in which the mutation loci of disorders such as PAHD and PCD were relatively abundant. A total of 10 types of mutations were found in PAH (OMIM *612,349), which accounted for 23.91% of all mutations, and the most common mutation c.852-855del in SLC25A13 (OMIM *603,859) accounted for 10.87% of all mutations.

**TABLE 4:** Mutations detected in patients with IEMs identified by tandem mass spectrometry (MS/MS).

Disorders (OMIM code)	Affected gene (OMIM code)	Nucleotide variant	Mutation alleles number	Relative frequency (%)	Portion of total mutations (%)
Phenylalanine hydroxylase deficiency (#261,600)	*PAH* (*612,349)		11		23.91
		c.721C > T	2	18.18	4.35
		c.208_210del	1	9.09	2.17
		c.826A > G	1	9.09	2.17
		c.969 + 2dup	1	9.09	2.17
		c.158G > A	1	9.09	2.17
		c.1174T > A	1	9.09	2.17
		C.728G > A	1	9.09	2.17
		c.440C > T	1	9.09	2.17
		C.498C > G	1	9.09	2.17
		5’UTR-exon1del	1	9.09	2.17
Neonatal intrahepatic cholestasis citrin deficiency (#605,814)	*SLC25A13* (*603,859)		8		17.39
		c.852-855del	5	62.50	10.87
		c.762T > A	1	12.50	2.17
		c.955C > T	1	12.50	2.17
		c.1638_1660dup	1	12.50	2.17
Citrullinemia type I (#215,700)	*ASS1* (*603,470)		3		6.52
		c.1048C > T	2	66.67	4.35
		c.649_651del	1	33.33	2.17
Tyrosinemia type III (#276,710)	*HPD* (*609,695)		4		8.70
		c.893A > C	1	25.00	2.17
		c.109T > G	1	25.00	2.17
		c.217T > C	1	25.00	2.17
		C.460G > A	1	25.00	2.17
Short-chain acyl-CoA dehydrogenase deficiency (#201,470)	*ACADS* (*606,885)		2		4.35
		c.1031A > G	2	100.00	4.35
Isovaleric acidemia (#243,500)	*IVD* (*607,036)		2		4.35
		c.433C > T	1	50.00	2.17
		c.865G > C	1	50.00	2.17
Methylmalonic aciduria combined with homocystinuria (#277,400)	*MMACHC* (*609831)		3		6.52
		c.457C > T	1	33.33	2.17
		c.481C > T	1	33.33	2.17
		c.482G > A	1	33.33	2.17
3-methylcrotonyl-CoA carboxylase deficiency (#210,200)	*MCCC1* (*609,010)		2		4.35
		c.1331G > A	1	50.00	2.17
		c.2035G > A	1	50.00	2.17
Primary carnitine deficiency (#212,140)	*SLC22A5* (*603,377)		9		19.57
		c.51C > G	2	22.22	4.35
		c.338G > A	1	11.11	2.17
		c.760C > T	2	22.22	4.35
		c.884 > T	1	11.11	2.17
		c.893C > T	1	11.11	2.17
		c.1340A>T	1	11.11	2.17
		c.1400C > G	1	11.11	2.17
Very-long-chain acyl-CoA dehydrogenase deficiency (#201,475)	*ACADVL* (*609,575)		2		4.35
		c.1280G > A	2	100.00	4.35

### Artificial Intelligence Disease Model Verification Results

A total of 1,988 cases were screened as positive for the first time, with a rate of 2.1%. However, the AI disease risk assessment model showed that out of the 94,684 analyzed samples, 1,058 samples were positive, with a rate of 1.12%. The number of samples with positive results concurrently indicated by the physician and the AI disease model was 614, while the number of samples with negative initial screening results (determined by the physician) and positive results determined by the AI disease model was 444. However, because some samples were at high risk for multiple IEMs (determined by the AI disease model), the total positive numbers for each IEM would be higher than the overall number of high-risk samples. Moreover, 21 confirmed cases of IEMs (TYR-III was not included in the panel of the AI disease model) were detected by the AI disease model that we screened from 2016 to 2020. The positive rates of each IEM are listed in Table 5.

**TABLE 5:** Comparison of interpretation results by AI and physician.

Disorders (OMIM code)	Positive by AI	Positive by physician	Positive by AI but first screening negative	Consistent rate between AI and physician (%)	Positive rate of AI disease model (%)	Positive rate of physician (%)	Reducing false-positive rate (%)
Primary carnitine deficiency (#212,140)	238	474	66	36.29	0.25	0.50	49.79
Methylmalonic acidemia (#251,000, #251,100, #251,110, #613,646, and #614,265), methylmalonic aciduria combined with homocystinuria (#277,400, #277,410, #277,380, #309,541 and #614,857), propionic acidemia (#606,054)	65	289	21	15.22	0.07	0.31	77.51
3-methylcrotonyl-CoA carboxylase deficiency (#210,200 and #210,210), beta-ketothiolase deficiency (#203,750), holocarboxylase synthetase deficiency (#253,270)	148	73	113	47.95	0.16	0.08	-102.74
Phenylalanine hydroxylase deficiency (#261,600), tetrahydrobiopterin deficiency (#233,910, #261,640, #612,716, #264,070, and #261,630)	131	83	64	80.72	0.14	0.09	-57.83
Short-chain acyl-CoA dehydrogenase deficiency (#201,470), isobutyryl-CoA dehydrogenase deficiency (#611,283)	139	78	79	76.92	0.15	0.08	-78.21
Hypermethioninemia (#250,850), homocystinuria (#236,200)	118	81	76	51.85	0.12	0.09	-45.68
Isovaleric acidemia (#243,500), 2-methylbutyryl-CoA dehydrogenase deficiency (#610,006)	96	111	36	54.05	0.10	0.12	13.51
Neonatal intrahepatic cholestasis citrin deficiency (#605,814), citrullinemia type I (#215,700), argininosuccinic aciduria (#207,900)	125	64	61	100.00	0.13	0.07	-95.31
Hyperprolinuria (#239,500)	6	14	6	0.00	0.01	0.01	57.14
Very-long-chain acyl-CoA dehydrogenase deficiency (#201,475)	38	28	10	100.00	0.04	0.03	-35.71
Carnitine palmitoyltransferase I deficiency (#255,120)	43	12	31	100.00	0.05	0.01	-258.33
Medium-chain acyl-CoA dehydrogenase deficiency (#201,450)	2	23	1	4.35	0.00	0.02	91.30
Ornithine transcarbamylase deficiency (#311,250)	15	192	3	6.25	0.02	0.20	92.19
Maple syrup urine disease (#248,600)	2	8	0	25.00	0.00	0.01	75.00
Glutaric acidemia I (#231,670)	10	45	3	15.56	0.01	0.05	77.78
Multiple acyl-CoA dehydrogenase deficiency (#231,680)	28	17	13	88.24	0.03	0.02	-64.71

*Negative numbers mean the efficiency of physician is better than the AI disease model.*

Of the 1,988 suspected positive cases, 579 were suspected of having IEMs that were not in the AI disease panel. Consequently, excluding the data of these 579 newborns, compared with the 1,058 suspected positives determined by the AI disease model, AI-based analysis of all metabolites decreased the rate of false positives by more than 24.9%. Without changing the sensitivity for detecting IEMs in the first screening, false-positive results were significantly reduced. There were 11 types of disorders that had significantly different determination results between the AI model and physicians: multiple acyl-CoA dehydrogenase deficiency (MCADD), OTCD, MMA, MMA-HCY, propionic acidemia (PA), GA-I, maple syrup urine disease (MSUD), PCD, 3-MCCD, β-ketothiolase deficiency (BKD), and holocarboxylase synthetase deficiency (HCSD; Table 5). The best performance of the AI disease model for IEMs was OTCD, which reduced the number of false-positives by 92.19%, followed by MCADD by 91.30%, GA-I by 77.78%, MMA, MMA-HCY, and PA by 77.51%, MUSD by 75%, and hyperprolinuria (H-PRO) by 57.14%.

We followed up the 444 samples, 157 samples were lost during a follow-up. In the follow-up results of the other 287 cases, we found one patient who had been previously diagnosed with NICCD in another hospital. Interestingly, the primary screening result of this sample was normal, which was detected in our laboratory once, with a citrulline test result of 31 μmol/L (a citrulline value of 6–32 μmol/L is considered normal). Analysis of this patient found two mutations in the *SLC25A13* gene (c.1638_1660dup and c.852_855del). This suggests that our AI disease model can be used as a complementary tool for MS/MS screening for IEMs to reduce the risk of missed conditions with current screening.

## Discussion

We screened 94,648 newborns and found that 22 cases were confirmed with IEMs, one additional case of NICCD was found by using the AI disease model, the incidence of IEMs in Shaoyang was 1:4,115, which was consistent with the rate reported in the United States (1:4,480) (19), but higher than in Japan (1:9,330) and Korea (1:13,205) (20). In China, the incidence of IEMs was reported to be higher in Suzhou (1:3,163) (21) and Quanzhou (1:2,804) (16) than what we found in the Shaoyang area. The incidence rates of IEMs vary greatly between countries and regions (the data are shown in Table 6).

**TABLE 6:** The incidence of different regions and countries.

Disorders (OMIM code)	Frequency						
	Shaoyang, China	Changsha, China	Suzhou, China	Quanzhou, China	Japan	Korea	United States
Screening numbers	94,648	300,849	401,660	364,545	3.36million	3.44million	11,750,876
**Amino acid disorders**	**1:7,281**	**1:10,745**	**1:5,084**	**1:8,680**	**1:26,000**	**1:29,000**	**1:12,648**
Phenylalanine hydroxylase deficiency (#261,600)	1:15,775	1:18,803	1:7,303	1:20,253	1:46,000	1:138,000	1:17,006
Citrullinemia type I (#215,700)	1:94,648	1:150,425	–	1:182,273	1:306,000	1:115,000	1:156,678
Neonatal intrahepatic cholestasis citrin deficiency (#605,814)	1:23,662	1:60,170	1:57,372	1:36,455	1:96,000	1:3,445,000	–
Tyrosinemia type III (#276,710)	1:47,324	1:300,849	1:200,830	–	–	–	–
**Organic acid disorders**	**1:31,549**	**1:25,071**	**1:13,389**	**1:9,347**	**1:22,000**	**1:31,000**	**1:18,682**
Isovaleric acidemia (#243,500)	1:94,648	1:150,425	1:200,830	1:91,136	1:672,000	1:138,000	1:139,891
Methylmalonic aciduria combined with homocystinuria (#277,400)	1:94,648	–	1:40,166	1:121,515	1:120,000	1:246,000	1:534,131
3-methylcrotonyl-CoA carboxylase deficiency (#210,200)	1:94,648	1:100,283	1:33,412	1:72,909	1:153,000	1:111,000	1:40,105
**Fatty acid oxidation disorders**	**1:13,521**	**1:9,705**	**1:9,129**	**1:7,440**	**1:30,000**	**1:111,000**	**1:11,034**
Primary carnitine deficiency (#212,140)	1:18,930	1:13,675	1:26,777	1:10,126	1:199,000	1:345,000	–
Short-chain acyl-CoA dehydrogenase deficiency (#201,470)	1:94,648	1:42,978	1:28,690	1:91,136	–	–	–
Very-long-chain acyl-CoA dehydrogenase deficiency (#201,475)	1:94,648	1:300,849	1:66934	1:121,515	1:93,000	1:383,000	1:57,043
Total	1:4,115	1:4,237	1:3,163	1:2,804	1:8,557	1:13,205	1:4,480

*The bold values mean all the IEMs are classified as amino acid disorders, organic acid disorders, and fatty acid oxidation disorders.*

The most common disorder among AADs was PAHD (26.09%), with an incidence rate of 1:15,775, which was higher than that reported by Lin et al. (16) in Quanzhou (1:26,039). Although c.158G > A and c.728G > A were reported to be the most common genetic mutations in *PAH* in the Chinese population (22), we identified 11 mutations with similar frequencies (23.91% of all mutations), with the most common mutation being c.721C > T, which was detected two times in Shaoyang (Table 4). NICCD was the second most common disorder in the region; four cases were detected (17.39% of all disorders), with an incidence rate of 1:23,662, which was higher than that found in Suzhou (1:57,372) and Quanzhou (1:36,455) (16). The mutation c.852-855del was the most common variant, accounting for 10.87% of all mutations in this region (Table 4), which is consistent with the results reported by Zhang et al. (23). Several studies have indicated that c.852-855del of the *SLC25A13* gene is a hotspot mutation in Chinese patients with NICCD, but without significant regional differences in the incidence rate.

Organic acid disorders were the least common IEMs in Shaoyang, and their incidence was lower than previously reported (16, 22). IVA, MMA, and 3-MCCD are uncommon in Japan, Korea, and other Asian regions, as reported by Shibata et al. (20). The incidence rates of IVA, MMA, and 3-MCCD in Japan were 1:672,000, 1:120,000, and 1:153,000, respectively, whereas in Korea, the rates were 1:138,000, 1:246,000, and 1:111,000, respectively (20).

Moreover, PCD was the most common FAOD in Shaoyang, with an incidence rate of 1:18,930. The prevalence of PCD is significantly different between races, with an incidence rate ranging from 1:37,000 to 1:100,000 in Australia and 1:142,000 in the United States (24–26). In the Faroe Islands, the incidence of PCD is significantly high with an incidence rate of 1:297 (27). In this study, we detected seven mutations in PCD-related genes in Shaoyang, though c.51C > G was the most common mutation in the *SLC22A5* (OMIM *603,377) gene, another hot-pot mutation c.1400C > G only accounted for 11.11%. There was a major difference with the frequencies of Suzhou (50%) and Ningbo (48.84%) (28).

Our research results were consistent with those of Peng et al. (8), who reported a random forest machine learning classifier on screening data to improve the prediction of true and false positives. In their analysis of the performance of the random forest machine, their model was able to reduce the number of false positives by 89% for GA-I, 45% for MMA, 98% for OTCD, and 2% for VLCADD. Although the performance of the random forest machine developed by Peng et al. (8) was better than that of our AI disease models for OTCD, GA-I, and VLCADD, the panel of our AI disease model included 27 disorders compared with the four disorders of their random forest machine model. Importantly, our AI model found one case of NICCD that was missed by physicians, which corroborates our AI disease model in terms of accuracy. NICCD is a type of citrin deficiency that is a hereditary IEM caused by *SLC25A13* mutations and manifests as neonatal intrahepatic cholestasis. An interesting report reveals that the carrier rate of *SLC25A13* mutations is 1:45 in the Chinese population, making it fairly common (14). However, in the traditional MS/MS screening program for IEMs, the total level of citrulline and its ratio to other metabolites are typically usually used for NICCD screening, but the sensitivity and accuracy in detecting NICCD are controversial. Elevated citrulline levels and ratios such as citrulline/glutamine and citrulline/arginine are not sensitive or efficient screening indicators. Our AI disease model had been trained with the data from missed cases in addition to positive confirmed cases, which enabled it to successfully identify true positive cases that were false negatives in primary screening.

In the strategies presented previously, new tests were generally added to reduce the occurrence of false negatives or false positives. For example, Lin et al. (14) proposed a strategy to incorporate genetic screening for NICCD into the current newborn screening program to reduce the occurrence of false negatives. Likewise, Monostori et al. (29) developed an assay using MS/MS for the simultaneous determination of the biomarkers 3-hydroxy propionic acid, methylmalonic acid, and methylcitric acid in neonatal dried blood spots. In contrast, we propose a novel strategy that combines MS/MS with AI in the current newborn screening program, which would be more efficient without the need for additional tests. The results of the AI analysis were generated quickly following primary screening.

In conclusion, expanded newborn screening by MS/MS does not always accurately detect IEMs. We demonstrate a novel proof-of-concept to optimize the newborn screening procedure, which combines expanded newborn screening with an AI disease model to identify IEMs and decrease the occurrence of false negatives and false positives. However, the AI disease model has its limitations. The performances for argininosuccinate aciduria (ASA), carnitine palmitoyltransferase I deficiency (CPT-ID), 3-MCCD, BKD, H-PRO, HCSD, and CIT-I were not satisfactory due to insufficient training data. Meanwhile, because some IEMs have common indicators, the AI model could not precisely identify these diseases. Hence, an AI model must be continuously trained, improved, optimized, and verified.

## Data Availability Statement

The original contributions presented in the study are publicly available. This data can be found here: Dryad Digital Repository, https://datadryad.org/stash/share/bT9bReq6IhkECKMvBqMgGkPNaT4NMqLPofz_7he4lFQ.

## Ethics Statement

The studies involving human participants were reviewed and approved by Ethic Committee of the Maternal and Child Health Hospital of Shaoyang City. Written informed consent to participate in this study was provided by the participants’ legal guardian/next of kin.

## Author Contributions

MZ: formal analysis, data curation, writing—original draft, and writing—review and editing. LD: editing and review and revision. YH: data curation. YX: methodology and validation. JW: conceptualization, project administration, supervision, and editing. NL: methodology and writing. YZ: review and editing, and supervision. HZ: software and validation. All authors contributed to the article and approved the submitted version.

## Conflict of Interest

The authors declare that the research was conducted in the absence of any commercial or financial relationships that could be construed as a potential conflict of interest.

## Publisher’s Note

All claims expressed in this article are solely those of the authors and do not necessarily represent those of their affiliated organizations, or those of the publisher, the editors and the reviewers. Any product that may be evaluated in this article, or claim that may be made by its manufacturer, is not guaranteed or endorsed by the publisher.

## Abbreviations

AADs: amino acid disorders
ASA: argininosuccinate aciduria
BH4D: tetrahydrobiopterin deficiency
BKD: beta-ketothiolase deficiency
CACTD: carnitine/acylcarnitine translocase deficiency
CIT-I: citrullinemia type I
CPT-ID: Carnitine palmitoyltransferase I deficiency
EE: ethylmalonic encephalopathy
FAODs: fatty acid oxidation disorders
GA-I: glutaric acidemia type I
H-ARG: arginemia
HCSD: holocarboxylase synthetase deficiency
HCY: homocystinuria
HHHS: hyperornithinemia-hyperammonemia-homocitrullinuria syndrome
H-MET: hypermethioninemia
H-ORN: hyperornithinemia
H-PRO: hyperprolinuria
H-TYR: tyrosinemia
IBDD: isobutyryl-CoA dehydrogenase deficiency
IEMs: inborn errors of metabolism
IVA: isovaleric acidemia
MADD: multiple acyl-CoA dehydrogenase deficiency.
MAL: malonic acidemia
MCADD: medium-chain acyl-CoA dehydrogenase deficiency
MMA: methylmalonic acidemia
MMA-HCY: methylmalonic aciduria combined with homocystinuria
MS/MS: tandem mass spectrometry
MSUD: maple syrup urine disease
NICCD: neonatal intrahepatic cholestasis citrin deficiency
OADs: organic acid disorders
PA: propionic acidemia
PAHD: phenylalanine hydroxylase deficiency
PCD: primary carnitine deficiency
SCADD: short-chain acyl-CoA dehydrogenase deficiency
TYR-III: tyrosinemia type III
VLCADD: very-long-chain acyl-CoA dehydrogenase deficiency
2-MBDD: 2-methylbutryl-CoA dehydrogenase deficiency
3-HMGD: 3-hydroxy-3-methylglutaryl-CoA lyase deficiency
3-MCCD: 3-methylcrotonyl-CoA carboxylase deficiency.
